# External radiation exposure, excretion, and effective half-life in ^177^Lu-PSMA-targeted therapies

**DOI:** 10.1186/s13550-018-0386-4

**Published:** 2018-04-12

**Authors:** J. Kurth, B. J. Krause, S. M. Schwarzenböck, L. Stegger, M. Schäfers, K. Rahbar

**Affiliations:** 1 Department of Nuclear Medicine, Rostock University Medical Center, Gertrudenplatz 1, 18057 Rostock, Germany; 2 Department of Nuclear Medicine, University Hospital Muenster, Muenster, Germany

**Keywords:** ^177^Lu-PSMA, Dose, Dose rate, Excretion, External radiation exposure, Wastewater

## Abstract

**Background:**

Prostate-specific membrane antigen (PSMA)-targeted therapy with ^177^Lu-PSMA-617 is a therapeutic option for patients with metastatic castration-resistant prostate cancer (mCRPC). To optimize the therapy procedure, it is necessary to determine relevant parameters to define radiation protection and safety necessities. Therefore, this study aimed at estimating the ambient radiation exposure received by the patient. Moreover, the excreted activity was quantified.

**Results:**

In total, 50 patients with mCRPC and treated with ^177^Lu-PSMA-617 (mean administered activity 6.3 ± 0.5 GBq) were retrospectively included in a bi-centric study. Whole-body dose rates were measured at a distance of 2 m at various time points after application of ^177^Lu-PSMA-617, and effective half-lives for different time points were calculated and compared. Radiation exposure to the public was approximated using the dose integral. For the estimation of the excreted activity, whole body measurements of 25 patients were performed at 7 time points.

Unbound ^177^Lu-PSMA-617 was rapidly cleared from the body. After 4 h, approximately 50% and, after 12 h, approximately 70% of the administered activity were excreted, primarily via urine. The mean dose rates were the following: 3.6 ± 0.7 μSv/h at 2 h p. i., 1.6 ± 0.6 μSv/h at 24 h, 1.1 ± 0.5 μSv/h at 48 h, and 0.7 ± 0.4 μSv/h at 72 h. The mean effective half-life of the cohort was 40.5 ± 9.6 h (min 21.7 h; max 85.7 h). The maximum dose to individual members of the public per treatment cycle was ~ 250 ± 55 μSv when the patient was discharged from the clinic after 48 h and ~ 190 ± 36 μSv when the patient was discharged after 72 h.

**Conclusions:**

In terms of the radiation exposure to the public, ^177^Lu-PSMA is a safe option of radionuclide therapy. As usually four (sometimes more) cycles of the therapy are performed, it must be conducted in a way that ensures that applicable legal requirements can be followed. In other words, the radiation exposure to the public and the concentration of activity in wastewater must be sub-marginal. Therefore, in certain countries, hospitalization of these patients is mandatory.

## Background

In recent years, the concept of “theranostics,” the effective combination of both imaging and treatment with radiopharmaceuticals using the same molecular target, has been successfully applied to imaging and treatment of castration-resistant prostate cancer (CRPC). CRPC is defined as disease progression despite an androgen-suppression therapy and is the most problematic stage of prostate cancer (PCa). The mean survival time for patients suffering from metastasized CRPC is approximately 1 to 2 years [1].

At present, the prostate-specific membrane antigen (PSMA) is the most widely characterized target antigen in PCa. PSMA is highly and specifically expressed on the surface of 90–100% of local prostate tumor cells, and in visceral and bone metastases at all tumor stages [2–5], and its expression increases with tumor aggressiveness [6, 7]. Therefore, PSMA has been proven to be an excellent target for prostate cancer imaging and therapy using ^68^Ga- and ^177^Lu-labeled radiopharmaceuticals, respectively [8].

A high-affinity PSMA ligand (PSMA-617) that shows an excellent tumor-to-background ratio, a rapid blood clearance, and that can be labeled with gallium-68, lutetium-177, or yttrium-90, was introduced by Benesova et al. [9]. Since then, a retrospective German multicenter study [10] also in addition to single-center studies, for example from Bad Berka [11], Bonn [12–14], Heidelberg [15, 16], Munich [17, 18] and Muenster [19–21], reported encouraging results for response rates after ^177^Lu-PSMA-targeted therapy. All studies showed that the therapy is well-tolerated. Hematological and renal parameters only changed insignificantly, and permanent xerostomia or other safety-related toxicity did not occur. The studies also suggest that the total number of therapy cycles is a positive predictor of the biochemical response. In addition, most importantly, the studies provide indications that ^177^Lu-PSMA-targeted therapy is associated with prolonged patient survival [20, 22, 23]. These promising results will probably lead to a significant increase in the use of PSMA-targeted therapies in the future. A good overview of the countries in Europe in which ^177^Lu-PSMA-targeted therapies are currently conducted is given by the recently published study by Sjögreen Gleisner et al. [24].

However, despite these promising findings, limitations must be critically discussed. As one example, nuclear medicine is always faced with consideration and minimization of the emission of radioactive residues and of the exposure of the population. Therefore, an increased number of therapies also requires a more intensive examination of the activity excreted by the patients and a conservative assessment of the dose which could be absorbed by the public and, in particular, by the relatives and caregivers.

It is known that most of the unbound ^177^Lu-PSMA-617 is excreted via the renal pathway with a high clearance rate [8, 17], which may lead to a contamination of public wastewater. In Germany, for instance, the limit for the discharge of lutetium-177 into municipal wastewater is 100 kBq/ml, according to the German Radiation Protection Ordinance [25]. It must also be considered that the incidence of prostate cancer is approximately 40 times higher than the incidence of neuroendocrine tumors (NET) [26–29], which will potentially increase the number of ^177^Lu-based therapies in the near future. The activity in municipal wastewater excreted by all patients could therefore reach a level that exceeds the legally defined limits. For diagnostic radiopharmaceuticals, theoretical values of the proportions of excreted activities can be derived using the information on biokinetic behavior in the body provided by report no. 128 of the International Commission on Radiological Protection (ICRP) [30]. However, for therapeutically used radiopharmaceuticals, these data can only be determined by direct measurements of the excreted activities.

In the 2007 recommendations of the ICRP and in the European Council Directive 2013/59/Euratom, the dose limit in planned exposure situations for the public is given as 1 mSv per year [31, 32]. A recently published study by Demir et al. aimed at investigating the radiation safety of a treatment protocol for ^177^Lu-PSMA therapy [33]. The researchers concluded that ^177^Lu-PSMA therapy is a safe treatment modality that can be applied as an outpatient protocol and that patients can be released from the radionuclide therapy ward approximately 6 h after administration of the therapeutic agent. However, a relatively high threshold of the dose rate of 30 μSv h^− 1^ at a distance of 1 m as release criteria was used, and no additional assessment of the excreted activity in relation to legal limits was performed. In contrast to this procedure in other countries, for instance, in Germany, Austria, or Italy, legal regulations demand hospitalization of these patients. In Germany, patients must be hospitalized for at least 48 h until the dose rate (measured at a distance of 2 m) is below a level warranting the dose to the public to stay below 1 mSv per year. According to the German directive “Radiation Protection in Medicine” [34] and the Recommendations of the German Commission on Radiological Protection [35], the procedure for the release of the patient is clearly defined: at the planned discharge of a patient from the therapy ward, the dose rate must be measured and documented individually. Furthermore, a mono-exponential reduction of the dose rate is assumed; thus, the dose to the public in the patient’s environment can be estimated by the time integral of the dose rate. The dose mainly depends on the distance to the patient and on the decrease of the residual activity in the patient, which may be described by the effective half-life.

A study by Fitschen et al. showed that the general framework mentioned above can be applied to peptide receptor radionuclide therapy (PPRT) of NET with ^177^Lu-Dotatoc/Dotatate [36]. If the effective half-life cannot be reasonably estimated from sequential measurements during the stay at the ward, the effective half-life is conservatively estimated to be equal to the physical half-life. However, this may prolong the stay in the hospital unnecessarily. Additionally, it should also be taken into account that in many patients, the therapy with ^177^Lu-PSMA is performed several times per year (normally up to 4 cycles). This will increase the dose to the public and, especially, to relatives and caregivers, as this group of persons is the most exposed category. Therefore, reliable measurements that form the basis to estimate and calculate these doses are necessary.

Consequently, the aim of this study was to give indications about the renal clearance of ^177^Lu-PSMA-617 through the quantification of the excreted activity with urine and to give an estimate of the radiation exposure to the public caused by patients treated with ^177^Lu-PSMA-617. We also aimed at investigating if it is possible to avoid the estimation of the patient-specific effective half-life during a therapy course by replacing it with a fixed half-life (e.g., the physical or another meaningful upper limit based on the mean effective half-life of a larger patient cohort).

## Methods

Results of measurements from two departments of Nuclear Medicine (Department 1: University of Muenster, Germany; Department 2: University of Rostock, Germany) were aggregated. Data from 50 randomly chosen patients, 25 from each center and treated with 1 cycle of ^177^Lu-PSMA-617, were included in the analysis. Table 1 summarizes the main characteristics of both cohorts.

**Table 1:** Main characteristic of the patient cohorts included in the analysis and time points of the dose rate and whole-body activity measurements

	Department 1	Department 2
Mean age	71.4 ± 9.2 years	70.3 ± 8.3 years
Mean activity	6.1 ± 0.5 GBq	6.6 ± 0.9 GBq
Time points *t*_*i*_ of the dose rate measurement	4, 24, and 48 h p. i.	2, 24, 48, and 72 h p. i.
Time points *t*_*j*_ of the whole-body activity measurement		Before the first bladder voiding and 2, 4, 12, 24, 48, and 72 h p. i.

All patients gave their written consent after being informed about possible side effects and risks of the therapy. Production and quality control of ^177^Lu-PSMA-617 were carried out according to the GMP regulations. The detailed labeling procedures were previously described by Ahmadzadehfar et al. [12]. The therapy was conducted in accordance with the German Medicines Law (AMG, §13[2b]) and the Consensus Recommendations of the German Society of Nuclear Medicine on ^177^Lu-PSMA therapy [18]. According to the German radiation protection regulations [34], all therapies were implemented as inpatient treatment and patients were hospitalized for at least 48 h.

The design of this study was presented to the ethics committee of the Rostock University Medical Center, and the need for a formal review was waived (file no. A 2017-0197). The retrospective and anonymized analysis was carried out in accordance with the Declaration of Helsinki and its later amendments and the legal considerations of clinical guidelines.

Lutetium-177 has a physical half-life of approximately 6.7 days and emits *β*^−^ particles (*E*_*β*_ of 497, 384, and 176 keV) and also *γ* photons with low energy (*E*_*γ*_ of 113 and 208 keV with low emission abundance of 6 and 11%, respectively) [37]. The therapeutic effect is mainly caused by the *β*^−^ component, while the emitted *γ* photons are generally used for the determination of bio-distribution. Therefore, we used external gamma and dose rate probes to carry out the necessary measurements. These types of measurements are robust, easy to perform, and implement into daily clinical routines and additional stress to the patients is minimized.

### Measurement of whole-body activity and excreted activity

The analysis of the excretion of unbound ^177^Lu-PSMA ligands and lutetium-177 was performed indirectly by measuring the remaining activity within the body of the patients at different time points with an external gamma probe. This has been proven to be a fast, simple, and robust method to estimate the activity within the body of the patient [38–41]. The difference compared with the expected activity that can be calculated directly by the physical decay of the administered activity is assumed to be equivalent to the excreted activity.

The measurements were carried out in Department 2, using a cohort of 25 patients (see Table 1). The whole-body activity was measured at 0 h (before voiding, reference) and approximately at 2, 4, 12, 24, 48, and 72 h after the administration of ^177^Lu-PSMA-617.

The remaining activity *A*_WB_ in the patient at the given time point *t*_*i*_ was determined by the following equation:

1 $$ {A}_{\mathrm{WB}}\left({t}_i\right)=\frac{\sqrt{C_A\left({t}_i\right)\cdot {C}_P\left({t}_i\right)}}{{\mathrm{CF}}_P}, $$

where *c*_*A*_(*t*_*i*_) and *c*_*P*_(*t*_*i*_) are the count rates (compensated for the background) at the given time point for the anterior and posterior views respectively. CF_*P*_ is a patient-specific calibration factor based on a baseline measurement of each patient, performed immediately after administration of ^177^Lu-PSMA-617 and before the first voiding of the bladder. This factor is calculated according to the following equation:

2 $$ {\mathrm{CF}}_P=\frac{\sqrt{C_A(0)\cdot {C}_p(0)}}{A_P}, $$

where *A*_*P*_ is the administered activity. This factor represents the efficiency of the detector for the spatially distributed activity and intrinsically considers the self-attenuation of the *γ* photons by the body of the patient.

The excreted activity *A*_ex_ at the time point *t*_*i*_ was calculated as the difference of the whole-body activity at the previous time point *t*_*i −* 1_ (corrected for decay) and the whole-body activity at the time point *t*_*i*_. The whole extracted activity can then be calculated as a summation:

3 $$ {A}_{\mathrm{ex}}={\sum}_{j=1}^i\left({A}_{\mathrm{WB}}\left({t}_{i-1}\right){e}^{-\frac{\ln (2)}{T_{1/2}}\bullet \left({t}_i-{t}_{i-1}\right)}-{A}_{\mathrm{WB}}\left({t}_i\right)\right), $$

with *T*_1/2_ representing the physical half-life of the radionuclide.

A 3″ × 3″-NaI (Tl) detector (Type 905-4) with multichannel analyzer and software (digiBASE, MAESTRO, Ortec, Oak Ridge, USA) was used as a measurement system. It was calibrated using americium-241, cobalt-57, cesium-137, and iodine-131 test sources, and regular quality checks were carried out. The shielded detector (collimator of 3 cm lead) was positioned 1 m above the floor, and the field of view covered the whole body of the patient. The energy window was set to 208 keV ± 27%, corresponding to approximately 3 × full width at half maximum of the energy resolution of the NaI (Tl) detector. The background was measured for 300 s without the presence of a patient. Patients were measured in anterior and posterior standing positions while holding arms close to the body at a distance of 2 m from the collimator surface. For all measurements, the acquisition time corrected for dead-time effects was set to 90 s. The observed dead-time effect during the calibration measurement for each patient resulted in a loss of detected counts of approximately 5%. No further corrections (e.g., for pile-up or non-linearity) were applied. Statistical uncertainties of the count rate were below 1% for all measurements, as recommended by Hindorf et al. [41]. Bi-exponential functions were fitted to the data, and the effective half-lives were calculated. The bi-exponential fit model was chosen after comparing the quality of fit of mono- and bi-exponential models using the Akaike criterion as described by Kletting et al. [42]. In addition, the results of dosimetry studies suggest that a bi-exponential fit would be appropriate [17, 43].

### Measurement of dose rate and estimation of dose to the public

The measurements and analyses were carried out with 50 patients from both departments as characterized in Table 1. All patients of Department 2 were released from the hospital 72 h p. i. for organizational reasons. Therefore, dose rate measurements were also available for *t*_*i*_ = 72 h p. i.

The determination of the expected dose *D* to the public requires knowledge of the dose rate DR at the time point of the release of the patient and of the effective half-life *T*_1/2eff_. The dose *D* can then be calculated according to the following equation:

4 $$ D=\frac{\mathrm{DR}\bullet {T}_{1/{2}_{\mathrm{eff}}}}{\ln 2}. $$

This equation was originally recommended by the German Commission on Radiological Protection to conservatively estimate the dose to the public after iodine-131 therapy [35]. However, as already mentioned, the study by Fitschen et al. showed that this equation is also applicable in PRRT [36].

In both departments, the dose rate monitor LB 123 D-H10 (Berthold Technologies, Bad Wildbad, Germany) was used, which is suitable for dose rate levels between 0.05 μSv/h and 10 mSv/h within an energy range from 30 keV to 1.3 MeV. The systems were officially calibrated, and regular quality checks were carried out. The patients were measured in the standing position from the anterior (DR_*A*_) and posterior (DR_*P*_) view at a distance of 2 m from the surface of the detector. The dose rate DR for different time points (see Table 1) was then calculated as the geometric mean of both measurements

5 $$ \mathrm{DR}\left({t}_i\right)=\sqrt{{\mathrm{DR}}_A\left({t}_i\right)\bullet {\mathrm{DR}}_P\left({t}_i\right).} $$

The data were fitted by a mono-exponential curve, and the effective half-life was calculated for the different time points: 24, 48, and 72 h (only from the data of Department 2). Since at least two data points are necessary for the fitting, the effective half-life was calculated from the following measurement points: 24 h (2 h/4 h and 24 h), 48 h (24 h and 48 h), and 72 h (24 h, 48 h and 72 h). For all comparisons, the time point 48 h was chosen as the reference, because it is standard that the patients are released from the therapy ward after 48 h. Subsequently, Eq. 4 was used to determine the dose *D* to the public after the discharge of the patient for different time points of release (24, 48, and 72 h p. i.), substituting *T*_1/2eff_ by *T*_1/2ind_ (individual half-life of each patient), *T*_1/2phys_ (physical half-life of ^177^Lu), and *T*_1/2max_ (maximum of the calculated effective half-life of the patient cohort) for different calculations. In addition, a correlation analysis (Spearman and Pearson) of the dose rate and administered activity was performed.

### Data processing and statistical analysis

Data processing and fitting were performed using in-house coded and validated LabVIEW applications (ver. 2016, Nat. Instruments), and SPSS (ver. 22.0, IBM Corp.) was used for the statistical analysis. Statistical differences were assessed using non-parametric tests for unpaired and paired samples (Mann-Whitney *U* test and Wilcoxon signed-rank test). A value of *p* ≤ 0.05 was assumed to indicate statistical significance. Data are presented as the mean and standard deviation unless stated otherwise.

## Results

### Whole-body measurement and analysis of excreted activity

**Fig. 1:**
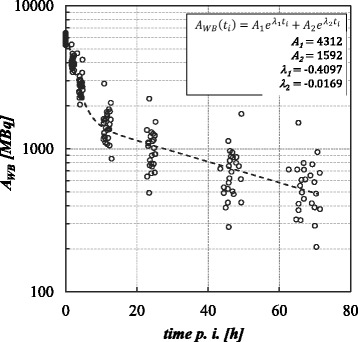
Time-activity curve of the measured whole-body activity and mean bi-exponential fit to the data

The measured time-activity curves (Fig. 1) revealed rapid clearance of the therapeutic agent from the body within the first few hours after infusion and a second slower phase. The calculated effective half-lives for the first and the second phase were 1.7 ± 0.8 h and 41.1 ± 9.3 h, respectively. No correlation was found between administered activity and measured half-lives (Pearson’s *r* = 0.08, Spearman’s rho = 0.07).

Table 2 summarizes the excreted activity of ^177^Lu-PSMA-617 for the different time points of measurement (calculated using Eq. 3) and the percentage of overall excreted activity. After approximately 4 h, approximately 50% and, after 12 h, almost 70% of the administered activities were excreted. Thereafter, the renal elimination decreased markedly. On average, approximately 4.8 GBq of the administered 6.6 GBq ^177^Lu-PSMA-617 were excreted over a period of 72 h.

**Table 2:** Excreted activity (*A*_ex_(*t*_*i*_)) between a given time point and the previous time point and the percentage comparison of the overall amount of excreted activity and administered activity

Time p. i.	2 h		4 h		12 h		24 h		48 h		72 h	
	*A*_ex_		*A*_ex_		*A*_ex_		*A*_ex_		*A*_ex_		*A*_ex_	
	[MBq]	[%]	[MBq]	[%]	[MBq]	[%]	[MBq]	[%]	[MBq]	[%]	[MBq]	[%]
Mean	1718	30	1265	50	1155	70	395	76	219	77	68	78
Std. Dev.	500	8	126	8	72	7	38	7	26	8	7	7
Min	735	13	1060	30	1009	48	318	53	160	50	50	54
Max	2621	42	1526	62	1323	82	474	87	265	89	82	89

### Measurement of dose rate and estimation of half-life and dose

**Fig. 2:**
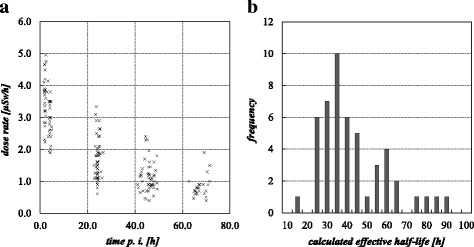
**a** Measured dose rates DR for all patients (*n* = 50). **b** Frequency distribution of the measured effective half-lives after 48 h

The measured dose rates at the different time points are summarized in Table 3, and Fig. 2a shows the course of the dose rate. Clearly, the drop of the dose rate is highest during the first 24 h, due to the fast excretion of the compound that can also be seen in the results of the first part of this study (see Fig. 1). Only a weak or no correlation between the measured dose rate and administered activity was found (Pearson’s *r* = 0.17; Spearman’s rho = 0.18).

**Table 3:** Measured dose rates and calculated effective half-lives at different time points

Time point	2 ± 0.3 h	3.9 ± 0.7 h	24.2 ± 0.7 h	45.7 ± 2.1 h	67.7 ± 2.3 h^a^
Dose rate [μSv/h] (measured at a distance of 2 m)					
Mean	3.6	2.8	1.6	1.1	0.7^a^
Std. Dev.	0.7	0.6	0.6	0.5	0.4^a^
Min	2.2	1.3	0.5	0.4	0.1^a^
Max	5.0	3.8	1.4	2.3	2.1^a^
Calculated effective half-life *T*_1/2eff_					
Mean	–	–	26.0	40.5	38.4^a^
Std. Dev.	–	–	12.4	15.8	9.6^a^
Min	–	–	11.0	21.7	25.7^a^
Max	–	–	73.5	85.7	74.5^a^

^a^These data are only available for 25 patients (cohort of Department 2)

Statistical tests of the calculated half-lives revealed no significant differences between the data of both cohorts (Mann-Whitney *U* test, *p* = 0.841 for 24 h and *p* = 0.883 for 48 h). Therefore, the datasets of both departments were aggregated for further analysis.

**Fig. 3:**
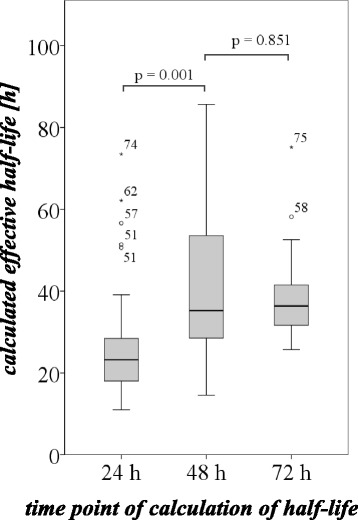
Calculated half-lives at different time points. The half-life at 24 h (calculated from the time points 2 or 4 and 24 h) is significantly underestimated, due to the influence of the first washout phase

The results for the calculated effective half-life at the different time points are summarized in Table 3. The histogram of the classified individual effective half-lives (calculated 48 h p. i.) for a class size of 5 h is shown in Fig. 2b. For most of the patients, a half-life between 35 and 40 h was calculated. In general, the results showed considerable variance (e.g., for the 48-h measurement point between 21.7 and 85.7 h, mean 40.5 ± 15.8 h) (illustrated in Fig. 3), and there were clear differences between the half-lives at the different measurement points. The calculated half-life after 24 h was significantly lower than after 48 h (Wilcoxon signed-rank test for paired samples, *p* = 0.016), caused by the influence of the initial rapid clearance of the compound (see the “Results” section in the paragraph above). Therefore, it seems inappropriate to use early time points of measurement to calculate the effective half-life and the dose to the public, as the calculated dose would be underestimated. No significant differences were found for the effective half-life after 48 and 72 h (Wilcoxon signed-rank test for paired samples *p* = 0.851). Therefore, in the sense of a conservative approach, we used the half-life of the slower elimination phase (calculated after 48 h) for further calculations of the dose *D*, as this is the main influence to the dose to the public.

The results for the calculated doses to the public after discharge of the patient using the three different types for the effective half-life are summarized in Table 4, which also adds a more detailed view of the minimum and maximum values.

**Table 4:** Calculated doses to the public for each cycle of ^177^Lu-PSMA-617 therapy for different time points of discharge of the patient from the hospital using different assumptions for the effective half-life

Estimated dose to the public [μSv] after 1 cycle of ^177^Lu-PSMA-617 therapy									
Time point of discharge	24 h p. i.^a^			48 h p. i.			72 h p. i.^b^		
Type of half-life	*T*_1/2ind_	*T*_1/2phys_	*T*_1/2max_	*T*_1/2ind_	*T*_1/2phys_	*T*_1/2max_	*T*_1/2ind_	*T*_1/2phys_	*T*_1/2max_
Mean	99	396	208	71	263	139	50	200	105
Std. Dev.	58	131	69	51	100	53	35	76	40
Min	25	142	75	13	93	49	17	93	49
Max	318	779	410	264	560	294	204	443	233

^a^For the effective individual half-life, the value from the 48-h measurement point was used (see the “Results” section)

^b^These data are only available for 25 patients (cohort of Department 2)

The expected dose to the public is below 1 mSv per cycle of therapy. When discharging the patient 48 h p. i., the expected dose is on average approximately 70 μSv with a maximum of approximately 260 μSv when the individual effective half-life is used for the calculation. Even in the case of an earlier release at 24 h p. i., the assumed dose to the public is on average 100 μSv with a of approximately 320 μSv per cycle.

If the physical half-life of lutetium-177 is used for the calculation (conservative approach), the estimated dose is still well below 1 mSv per cycle. However, the average dose to the public will be overestimated by approximately 350%, in some cases by up to 640%, as the individual half-life is much lower than the physical half-life of lutetium-177. The use of the maximum half-life of our cohort *T*_1/2max_ of 85 h results in a conservative dose estimate that is reduced by approximately 90% compared to the use of the physical half-life.

## Discussion

In the relatively short time since entering the clinical arena, PSMA-targeted therapies have shown promising results and a further intensification of their use is to be expected. Due to the increasing number of radionuclide therapies, radiation protection issues must also be taken into account when optimizing therapy protocols. In the work presented here, we describe the results of some essential aspects of radiation protection in the context of ^177^Lu-PSMA therapy, namely, the exposure of the general population by treated patients and the excretion of unbound or metabolized radioactive PSMA compounds.

Unbound ^177^Lu-PSMA-617 is mainly excreted through the renal pathway with a fast renal clearance, due to the low molecular weight of the compound [9]. As known from PRRT of NET, the amount of activity in the excreted feces and perspiration is also negligible for PSMA ligands [8, 9]. Our results show the two phases of excretion; a faster initial clearance with a half-life of approximately 2 h and a much slower clearance of approximately 41 h, also qualitatively described by Kulkarni et al. and Hohberg et al. [11, 44] and known from the PRRT of NET [45, 46]. The clearance of ^177^Lu-PSMA-617 is faster than the clearance known from ^177^Lu-Dotatoc/Dotatate (effective half-life approximately 56 h) [36, 45]. Our results show that within the first 12 h after the administration of ^177^Lu-PSMA-617, the majority of the administered activity is excreted (approximately 70 ± 7%). These results are comparable to those known from PPRT of NET with ^177^Lu-Dotatoc/Dotatate, as studies by Esser et al. and Calais et al. have shown that after 6 h, nearly 45 to 50% and, after 12 h, approx. 65 to 70% of Dotatoc/Dotatate compounds are excreted by urine [46, 47]. Furthermore, a recently published study by Demir et al. also analyzed the excreted activity in a group of 7 patients treated with ^177^Lu-PSMA-617 [33]. The researchers observed that a mean of 45% (range, 32–65%) of the administered activity is excreted within the first 6 h, which is somewhat lower but comparable to our results. However, no results for the following days and the overall amount of excreted activity are reported.

To determine the renal excreted activity, the activity in the urine is usually measured. By contrast, we opted for the indirect determination of excreted activity, because the measurements of the specific activity in the collected urine showed several disadvantages in clinical routine. Some of the disadvantages include incomplete urine collection, contamination, and an increased patient stress, owing to the fact that many patients suffering from mCRPC also suffer from a strong urge to urinate, frequent urination, or incontinence. Therefore, we determined the excreted activity on the basis of measurements of whole-body activity at different time points using an external gamma probe. A potential source of uncertainty discussed in the context of activity determination by whole-body measurements is the varying activity in the bladder during the baseline measurement and differences of the activity redistribution in the patient body during subsequent measurements. A recently published study by Liu et al. compared the results of the urine collection method and whole-body measurements in patients treated with ^177^Lu-Dotatate [48]. They found that the excreted activities determined by whole-body measurements were overestimated by approximately 14% at 1 h p. i. and randomly varied from − 29 to 49% at 24 h. The authors also proposed a modified setup of the whole-body measurements: a series of paired measurements before and after each voiding of the bladder to correct for the effects of the varying activity distribution. However, it was not possible in our cohort of patients to establish this comparatively extensive measuring regimen, since many patients felt a strong sudden urge to urinate. Due to that, the individual reference measurements, which are necessary before each use of the toilet, often could not be acquired by the technicians, and thus, the necessary correction factors could not be calculated. On the other hand, a study on dosimetry for ^177^Lu-PSMA-617 therapies by Hohberg et al. [44] showed only minor deviations between whole-body activities measured by external gamma probes and the activities measured by planar whole body gamma camera imaging; planar whole body scintigraphy is assumed to be the most accurate method in this case. The whole body doses calculated from the resulting time-activity curves also showed only minor differences (≤ 2.36 ± 1.69%). Based on this result, we decided to use the conservative and well-known approach of measuring whole-body activity using external gamma probes.

On average, 4.81 GBq and maximally up to 6.29 GBq of lutetium-177 are excreted per therapy cycle and patient (see Table 2) based on an average therapeutic activity of 6.6 GBq in patient cohort 2. Due to the high incidence of prostate cancer, this will certainly lead to an increased concentration of lutetium-177 in wastewater. Particular attention must be paid in compliance with legal and local regulations and statutory thresholds where applicable. It should also be noted that the incidence of prostate carcinoma, and thus the number of expected therapies, is considerably higher than the incidences we know from NET therapies. Furthermore, experience from the NET therapies with ^177^Lu-Dotatoc/Dotatate have shown that, differing from the standard therapy regimen (e.g., the NETTER 1 trial, 4 × 7.4 GBq) [49], also, more than 4 cycles per patient are tolerated very well. This was demonstrated by a recently published study by Yordanova et al. [50], for example, in which up to 13 cycles of PRRT with a median activity of 63.8 GBq were administered without severe toxicity. This finding is also to be expected for PSMA-targeted therapies; therefore, the amount of wastewater contaminated with lutetium-177 will most likely increase. When balancing the quantities of liquid waste of a nuclear medicine ward, the excreted activities of other therapies also have to be considered. If, for instance, both PSMA and Dotatoc/Dotatate therapies are performed, an excretion factor of 90% can be used independently for both therapies to assess the excreted activity. This conservative value takes into account both the results of studies on excretion in Dotatoc/Dotatate therapies [46, 47] and the results published by Demir et al. [33] and our results on PSMA therapies.

The results for dose rate measurement and estimation of dose to the general public are not as critical. Our test results are based on measurements at a distance of 2 m according to legal requirements in Germany. The fast component of the kinetics, with a half-life of 1.7 h, does not provide any relevant contribution to the dose of the general public. The mean value determined for the effective half-life is approximately 41 h in ^177^Lu-PSMA-617-targeted therapies. The calculated doses to the public show great variations. However, it can be clearly seen from Tables 1 and 4 that, if the individually calculated half-life can be used for dose estimation, for the majority of the patients, the dose limit for the general public of 1 mSv/year will not be reached even in the case of several therapy cycles per year, and also, no adjustments of the hospital stay are necessary. However, the dose limits are exceeded in some patients, especially in patients with a high tumor load or impaired kidney function and in cases of early discharge and more than two therapy cycles per year. If it is not possible to perform the necessary measurements to calculate the individual half-life, for instance, due to the poor health status of the patient, the use of the maximum effective half-life of 85.7 h for the dose estimation is a good alternative as already proposed by Fitschen et al. for ^177^Lu-Dotataoc/Dotatate therapies [36]. If the physical half-life of lutetium-177 is used instead, the dose to the public will be overestimated by several hundred percent (see Table 4). However, this approach is too conservative and may lead to an unnecessary prolongation of the hospital stay. However, if more cycles of PSMA-targeted therapy per year and patient are conducted, the estimated dose to the public for each cycle should be summed to an individual annual dose. If this result shows that the limit of 1 mSv might be exceeded, the patient’s stay in the hospital can be adjusted accordingly.

The safety of ^177^Lu PSMA administration to staff and caregivers was also emphasized in the previously cited study by Demir et al. [33]. The authors measured radiation doses delivered both to administering staff and family in 23 patients and found that the mean dose rate at 1 m after 4 and 6 h was 23 ± 6 μSv/h and 15 ± 4 μSv/h, respectively. They also found that the mean dose received by close family members was 202 ± 43 μSv, measured for 5 days post-injection with an optically stimulated luminescence dosimeter. These doses are slightly higher than those calculated in our study; however, the administered activity was higher (7.5 GBq compared to 6.3 GBq), and all of the patients were released from the hospital 6 h p. i. Both factors increase the dose rate, and therefore, the dose received by the close family members would be higher. In summary, the results show that the equation used in our study (Eq. 4) provides a good estimation of the dose to relatives and caregivers.

When interpreting the results of our study with regard to dose limits, any differences between the general population and relatives that may have been made by legal regulations must be taken into account. For instance, in the German Radiation Protection Ordinance and in the European Council Directive 2013/59/Euratom, no difference is made between the group of relatives and caregivers and the general public; for both groups, the dose limit is 1 mSv per year [25, 32]. The ICRP recommends a dose limit of 1 mSv per year for the general public and up to 5 mSv per year for relatives and caregivers [31]. It must also be noted that the results of our study are based on patients treated with ^177^Lu-PSMA-617. However, comparable results can be expected also for other PSMA ligands, such as PSMA-I&T because these ligands do not significantly differ in biokinetics [11].

In general, the success of therapies with Dotatoc/Dotatate and PSMA ligands will potentially lead to an increase of radionuclide therapies with lutetium-177 in clinical routine, because at present, these therapies have also shown promising results with respect to response rates with only minor side effects [21, 49, 51]. Promising new targets for the concept of theranostics are on the horizon, such as the chemokine receptor 4 (CXCR4) [52], the gastrin releasing peptide receptor [53, 54], and integrin α_v_β_3_ [55]. Therefore, it is certainly not an exaggeration to state that we will see an increased use of ^177^Lu-labeled therapeutics in the near future. This aspect also has to be taken into account when therapy protocols and the necessary radiation protection measures (inpatient or outpatient treatment, time point of discharge, etc.) are planned. In this context, the treatment of wastewater from nuclear medicine therapy wards and their compliance with the legal regulations must also be considered. For instance, a series of simple screening techniques that can be used to demonstrate compliance with administratively set reference levels for the release of radionuclides is provided by report no. 123 of the National Council on Radiation Protection and Measurements (NCRP) [56]. If there are doubts about compliance with legal limits, the hospitalization of the patients at a therapy ward with an appropriate decay tank is an option if this is not already prescribed by law, as it is in Germany.

## Conclusions

Due to the expected increase in PSMA-targeting therapies, radiation protection with regard to the exposure of the general public and the excretion of free or metabolized activity must be considered. In case of the appropriate adaptation of the discharge time point, it is possible to comply with the recommended dose limit of 1 mSv per year for the general public according to ICRP 103, even in the case of performing several therapy cycles per calendar year and patient. If the effective half-life cannot be determined individually, a maximum value of approximately 85 h can be used instead of the physical half-life of lutetium-177 to conservatively calculate the expected dose to the general public. However, special attention must be paid to the activity excreted with the urine. Depending on local conditions (e.g., the number of treated patients, amount of wastewater), the activity concentration in the wastewater will potentially have to be taken into account, especially in light of the rising numbers of ^177^Lu-based therapies.

## Abbreviations

ICRP: International Commission on Radiological Protection
mCRPC: Metastasized castration-resistant prostate cancer
NCRP: National Council on Radiation Protection and Measurements
NET: Neuroendocrine tumor
PRRT: Peptide receptor radionuclide therapy
PSMA: Prostate-specific membrane antigen
